# The association between different outcome measures and prognostic factors in patients with neck pain: a cohort study

**DOI:** 10.1186/s12891-022-05558-5

**Published:** 2022-07-14

**Authors:** Birgitte Lawaetz Myhrvold, Alice Kongsted, Pernille Irgens, Hilde Stendal Robinson, Nina K Vøllestad

**Affiliations:** 1 Department of Interdisciplinary Health Sciences, Institute of Health and Society, University of Oslo, Blindern, P.O. Box 1089, 0317 Oslo, Norway; 2 Department of Sports Science and Clinical Biomechanics, University of Southern Denmark, Odense, Denmark; 3 Chiropractic Knowledge Hub, Odense, Denmark

**Keywords:** Neck pain, Prognostic factors, Prediction, Outcome measures, Pain intensity, Neck disability index, Health-related quality of life

## Abstract

**Background:**

Health domains like pain, disability, and health-related quality of life are commonly used outcomes for musculoskeletal disorders. Most prognostic studies include only one outcome, and it is unknown if prognostic factors and models may be generic across different outcomes. The objectives of this study were to examine the correlation among commonly used outcomes for neck pain (pain intensity, disability, and health-related quality of life) and to explore how the predictive performance of a prognostic model differs across commonly used outcomes.

**Methods:**

We conducted an observational prospective cohort study with data from patients with neck pain aged 18–84 years consulting Norwegian chiropractors. We used three different outcomes: pain intensity (Numeric Pain Rating Scale), the Neck Disability Index (NDI), and health-related quality of Life (EQ-5D). We assessed associations between change in outcome scores at 12-weeks follow-up with Pearson’s correlation coefficient. We used multivariable linear regression models to explore differences in explained variance and relationship between predictors and outcomes.

**Results:**

The study sample included 1313 patients and 941 (72%) completed follow-up at 12 weeks. The strongest correlation was between NDI and EQ-5D (r = 0.57) while the weakest correlation was between EQ-5D and pain intensity (r = 0.39). The correlation between NDI and pain intensity was moderate (r = 0.53) In the final regression models, the explained variance ranged from adjusted R^2^ of 0.26 to 0.60, highest with NDI and lowest with pain intensity as outcome. The predictive contributions of the included predictors were similar across outcomes. Among the investigated predictors, pain patterns and the baseline measure of the corresponding outcome measure contributed the most to explained variance across all outcomes.

**Conclusions:**

The highest correlation was found between NDI and EQ-5D and the lowest with pain intensity. The same prognostic model showed highest predictive performance with NDI as outcome and poorest with pain intensity as outcome. These results suggest that we need more knowledge on the reasons for the differences in predictive performance variation across outcomes.

**Supplementary Information:**

The online version contains supplementary material available at 10.1186/s12891-022-05558-5.

## Background

Patients seeking health care for chronic musculoskeletal (MSK) pain report problems with a variety of everyday life health domains that includes physical activity, vitality, mental well-being, sleep, work, and social relationships [1–4]. They also report a wide range of goals for treatment, and it is shown that goals set by patients with low back pain included a mixture of health domains [3, 5, 6]. In response to these challenges, a set of core outcome measures covering a range of health domains including pain, disability, and health-related quality of life (HRQoL) have been suggested [7–9]. Studies that investigate the association between outcome measures report only a fair to moderate relationship, this indicate dissociations between pain, disability and HRQoL [10–12]. Despite this, most prognostic studies define one single primary outcome and patients are evaluated on the same outcome dimension irrespective of their treatment goals and expectations.

A large variety of individual prognostic factors have been identified with improvement in patient with neck pain [13–18]. Age, pain intensity, disability, previous neck pain history, widespread pain and expectations all have an individual association with pain intensity, disability, and global perceived change as outcomes [14–18]. The same prognostic factors have all been tested for inclusion in prognostic models where the focus is not the individual predictor effect but rather the performance of a combination of prognostic factors as a whole [19]. We recently externally validated and updated an existing prognostic model for patients with neck pain [20]. The model includes seven predictors and was developed to predict improvement after 12 weeks using Global perceived effect (GPE) as outcome measure. However, it is unknown how well one single outcome measure will capture the breath of goals reported by patients with neck pain. Few studies have investigated if the predictive performance of prognostic factors or models varies across outcomes representing different health domains [19]. In existing prognostic model studies, a pre-set of candidate prognostic factors are defined and subsequently for each outcome different prognostic models are developed, and the predictive performance of each model reported [21, 22]. Studies rarely explore if predictors included in a model equally predict outcomes that represent different health domains (predictive strength), and how well a model predicts different outcome measures (predictive ability). It is likely that the predictive strength of a prognostic factor or the predictive ability of a model will differ depending on the construct and health domain of the outcome measure selected. As patients may pursue treatment goals related to diverse outcomes including pain, disability and HRQoL, clinicians need to know to what extent prognostic factors and prognostic models relate to each of these. Thus, to explore prognostic factors and models regarding their predictive performance for outcomes that measure different health domains may provide a more comprehensive picture of prognosis and insights into how well the predictions match the wide range of patients' goals.

The aims of this study were to (1) examine the association among commonly used outcomes for neck pain (i.e., pain intensity, disability and HRQoL), (2) investigate if the predictive ability of a recently developed prognostic model for GPE of neck pain differs across outcome measures (i.e., pain intensity, disability and HRQoL), and (3) explore the predictive strength of the included predictors across outcome measures.

## Methods

### Study design and setting

This study was part of a one-year prospective observational cohort study that aimed to identify prognostic factors for neck pain patients in chiropractic practice in Norway [20]. The study was reported according to the STROBE statement [23].

The study was approved by The Norwegian Regional Committee for Medical and Health Research Ethics (2015/89).

### Recruitment of patients and study samples

Chiropractors invited consecutive patients with neck pain to participate in the study from September 2015 till May 2016. The chiropractors (*n* = 71) were located across Norway representing both urban and rural settings. Prior to inclusion, patients received oral and written information about the study from the chiropractor. We invited all patients presenting with neck pain as a primary or secondary complaint to participate. The participants were included regardless of neck pain classification, pain duration and time since last chiropractic consultation or treatment. Thus, the neck pain could be a first episode or part of an episodic or persistent pain complaint. Inclusion criteria were as follows: age above 18, adequate understanding of Norwegian language to complete questionnaires, own and be able to operate a mobile phone. Exclusion criteria were as follows: no serious pathologies such as suspected inflammatory disorders, fractures, infection, malignancy, or nerve root involvement requiring referral to surgery. In addition, we asked the included chiropractors to report the reason why a patient did not want to participate or was not invited in the study. We attempted to contact all non-responders by phone and/or mail in order to collect information on the reason for the drop out. Participants signed a written consent.

### Measurements

The data collection included self-reported questionnaires at baseline and after 12 weeks as described in detail previously [20]. Treatment was not affected by study participation. We chose follow-up at 12 weeks as endpoint [24–26].

### Patient-reported baseline information

We included predictors from our previous external validation and update of a prognostic model developed by Schellingerhout and colleagues [20, 27]. The predictors of the updated model were pain patterns of neck pain the previous year and expected pain patterns of neck pain the upcoming year (described below), radiating pain to the shoulder and/or elbow (yes/no), number of MSK pain-sites (0–10) and educational level (low, medium and high). We measured the physical leisure activity predictor as a 5-point ordered scale (Never/Less than once a week/Once a week/2–3 time a week/More than 3 times a week). In order to identify a difference between no activity versus activity we categorized the physical leisure activity predictor into ‘doing activity once or more per week’ versus ‘doing activity never or less than once per week’ (≥ 1 per week/ < 1 per week) [20]. The included predictors represent a variety of known and well-documented demographic and psychosocial prognostic factors that reflects the different aspects of health domains [20].

Pain patterns were measured by a self-reported visual trajectory pattern questionnaire [20]. The questionnaire had descriptions of five different patterns of neck pain that aim to characterize patients’ neck pain the past year (Previous pattern) or expectations of neck pain the upcoming year (Expected pattern) [20]. The five pain patterns were based on existing literature on trajectory patterns [28]. These pain patterns illustrate an increasing severity from the Single pain episode to the Severe ongoing pain pattern [29, 30].

### Clinician-reported baseline information

The chiropractors recorded the consultation-type, i.e. when in the clinical course of treatment, participants were recruited: “First-time consultation” described patients recruited at the first visit, “Follow-up consultation” described patients recruited during a clinical course of treatment, and “Maintenance consultation” described patients visiting the chiropractor regularly at pre-planned time points [31].

### Outcomes

The outcome measures covered the health domains of pain intensity, disability, and HRQoL.

Neck pain intensity was measured by a numeric rating scale (NRS) rating from 0 indicating ‘No pain’ to 10 indicating ‘worst pain imaginable’ [32]. We used the score at week 12 as pain intensity outcome. The NRS scale has been shown to have good test–retest reliability, construct validity and responsiveness for pain intensity [32].

The Neck Disability Index questionnaire (NDI) was used to assess disability [33]. It consists of 10 items evaluating function, pain, sleep quality and work ability, each scored from 0–5, with a sum-score range of 0 to 50 points. A higher score indicates more disability. The NDI has been reported to be a reliable, valid and responsive outcome measure in various neck pain populations, including different neck pain conditions [34–36].

EQ-5D was used to assess the HRQoL [37]. It evaluates 5 dimensions; mobility, self-care, usual activities, pain/discomfort, and anxiety/depression. We used the version with a 5-level response and the scoring algorithm from the United Kingdom to calculate a health state index ranging from 0 (equivalent to being dead) to 1 (full health). EQ-5D was developed to be relevant to a wide range of health conditions [37] and has been reported to be a reliable and valid outcome measure for neck pain [38, 39]. Patient participation was not blinded, as all outcome measures and prognostic factors were patient-reported. However, patients completed all questionnaires independently and in absence of the chiropractor or researchers.

### Statistical analysis

We present patient characteristics and outcomes as frequencies with percentages, means and standard deviations (SD). The outcome change score is presented by median with interquartile range (IQR) for pain intensity, NDI and EQ-5D. The statistical difference between baseline and follow-up measurements were tested by paired t-tests for all outcome measures.

We assessed the association between change score of the outcome scales at 12 weeks with Pearson’s correlation coefficient (r). The strength of r was interpreted according to the coefficient values < 0.3 (weak), 0.3 to 0.7 (moderate) and >  = 0.7 (strong) [40, 41]. In addition, we used Lowess plots to visualize the relationship between pairs of change score of the outcomes. For each outcome, we determined effect sizes by dividing the mean change between baseline and 12-week follow-up, by the SD of the baseline score. Cohen’s guidelines define an effect size of about 0.1 as small, 0.3 as medium, and 0.5 or higher as large [42].

We conducted a series of multivariable linear regression analyses using block-wise entry to investigate the predictive performance of individual predictors and model for each outcome measure. We divided the selected predictors into four blocks based on assorted health domains used when updating the prognostic model [20]. First, we entered each single block (Block 1 to 4) into the model and we determined their isolated contribution to prediction. Thereafter, we sequentially combined the blocks in four steps, and determined the combined contribution to prediction of each model. The final model comprised all 4 blocks. Block 1 included the pain patterns to account for previous and expected pain symptoms; block 2 included radiating pain and number of MSK pain-sites to account for additional pain history; block 3 included education level, physical leisure activity and the interaction term ‘physical leisure activity#number of MSK pain-sites’ to account for sociodemographic variables. Block 4 included consultation-type.

To clarify the predictive contribution of the baseline value of outcomes for each outcome measure, we formed a fifth block (Block 5). Block 5 included the baseline value of either pain intensity (Block 5a), NDI (Block 5b) or EQ-5D (Block 5c). Separately, we combined each block to the final model (i.e., Block 5a to the final model; Block 5b to the final model; Block 5c to the final model). In addition, we investigated the individual predictive performance of each of the three blocks, Block 5a, Block 5b and Block 5c.

We report the predictive performance as predictive ability and strength of association. For the predictive ability the adjusted R^2^, i.e. the proportion of the variance in outcome explained by the prognostic factors, was compared across models. Furthermore, we report the relationship between predictors and outcomes as the strength of association (beta coefficients with 95% confidence intervals (CIs)). In all regression analyses, we transformed both baseline outcome variables and outcomes to a continuous scale of 0–100, i.e. (score/max of scale score) × 100, to allow comparison of model explanation and strength of association across outcomes. We assessed the normality assumptions for linear regression visually for each model based on the residual plots and Q-Q plots. We investigated the linear association between predictors and outcomes by added-variable plots. The amount of missing data was small and no predictor had more missing values than 2.9%. We assumed missing values were due to random processes, as the main reason for missing were mostly incomplete paper-based questionnaires, and not due to refusal of patients or chiropractors to fill inn questionnaires. For missing values of the predictors, we used the multiple imputation method. For each outcome, we examined if the available sample size was enough for exploring prognostic models [43]. We used the method by Riley et al. to calculate for the efficient sample size for multivariable linear regression modeling [44]. In the present study, seven candidate predictors (that included 18 parameters) were selected a priori based on an updated prognostic model for neck pain [20]. We pre-specified the anticipated R^2^ (0.8), and used mean and standard deviation of outcomes in this study sample. This specifies a sample size of 254 is required. Our total sample size included 941 patients and within acceptable limits. We set the significance level at 5% for all tests and performed all analyses in STATA/SE 16 (STATA Corp, College Stations, TX).

## Results

Baseline data from 1313 patients were collected of whom 941 (72%) responded to 12 weeks follow-up and constituted the study sample used for analyses in this study (see flowchart in Additional file 3). Study participants and non-responders (*n* = 372, 28%) were comparable in terms of demographics, neck pain symptoms and history, general health and psychosocial factors with only minor and not very substantially differences observed (Table 1).

**Table 1:** Baseline characteristics of the study population (*n* = 941) and Non-responders at 12-week follow-up (*n* = 372)

	**Study sample**	**Non-responders**
Number participants, n	941	372
**Sociodemographic and physical leisure activity**		
**Gender,** females (%)	693 (74)	274 (74)
**Age**, mean years (SD), Range (18–85)	45 (13)	41 (13)
**Education level**, n (%)		
High	535 (57)	172 (46)
Medium	357 (38)	177 (48)
Low	48 (5)	20 (5)
**Physical activity,** ≥ 1 per week (%)	652 (69)	256 (69)
**Employment status**, employed (%)	761 (81)	301 (81)
**Clinician-reported**		
**Consultation-type**, n (%)		
First-time consultation	145 (16)	76 (21)
Follow-up consultation	271 (30)	111 (31)
Maintenance consultation	498 (54)	171 (48)
**Neck pain symptoms and history**		
**Radiating pain to shoulder and/or elbow**, yes (%)	707 (75)	279 (75)
**Patient Previous pattern**, n (%)		
Single episode	88 (9)	33 (9)
Episodic pain	307 (33)	105 (28)
Mild pain/ recovering	87 (9)	36 (10)
Fluctuating pain	404 (43)	161 (43)
Moderate/severe pain	23 (2)	15 (4)
Neither/ Unsure	24 (2.5)	14 (4)
**Pain intensity**, (0–10), mean (SD)	4.7 (2.2)	4.9 (2.3)
**Previous episodes**, n (%)		
No, first	125 (13)	61 (16)
Yes, 1–3 times previously	147 (16)	73 (20)
Yes, > 3 times previously	241 (26)	87 (23)
Yes, More or less chronic	425 (45)	151 (41)
**Duration current episode**, n (%)		
< 1 month	206 (22)	92 (25)
1–3 months	133 (14)	57 (15)
> 3 months	591 (63)	216 (58)
**NDI**, (0–50) mean (SD)	11.5 (6.6)	12.1 (6.8)
**General health/Comorbidity**		
**Number of MSK pain-site**, (0–10), mean (SD)	4.6 (2.2)	4.6 (2.2)
**EQ-5D-index,** (0–1), mean (SD)	0.85 (0.13)	0.85 (0.12)
**Psychosocial**		
**Patient Expected pattern**, n (%)		
Single episode	191 (20)	68 (18)
Episodic pain	331 (35)	119 (32)
Mild pain/ recovering	108 (11)	45 (12)
Fluctuating pain	191 (20)	88 (24)
Moderate/severe pain	5 (0.5)	6 (2)
Neither/ Unsure	114 (12)	45 (12)
**Expectations**, mean (SD)	5.9 (3.1)	5.6 (3.1)
**HSCL**, (0–4), mean (SD)	1.6 (0.5)	1.7 (0.5)
**Örebro screening questionnaire**, (0–100), mean (SD)	39.5 (15.4)	40.9 (16.2)

Pain intensity from NRS: the 11-point numerical rating scale [32], psychosocial risk factors: Örebro Screening Questionnair [54, 55], *HSCL-10,* Hopkins Symptom Checklist measuring emotional stress [56], *NDI,* Neck Disability Index measuring disability [33], EQ-5D-index measuring HRQoL [37], *SD,* Standard deviation

### Outcome change score and correlations

There were small to moderate improvements for all outcomes from baseline to 12 weeks (P < 0.001). The mean (SD) pain intensity decreased from 4.7 (2.4) to 2.7 (2.1), NDI decreased from 11.5 (6.6) to 9.4 (6.4) and EQ-5D utility score increased from 0.85 (0.13) to 0.88 (0.11). The median (IQR) for the outcome change scores were -2 (-4 to 0), -2 (-4 to 1) and 0.01 (-0.02 to 0.05), respectively.

There was, however, a large inter-individual variation in changes score as illustrated with Lowess plots (Additional file 1). The plots and the Pearson correlation coefficients between change score on the outcome scales at 12 weeks revealed the strongest correlation between NDI and EQ-5D (r = 0.57) and between NDI and pain intensity (r = 0.53), while the weakest correlation was between EQ-5D and pain intensity (r = 0.39). The effect sizes for pain intensity, NDI and EQ5D were 0.76, 0.46 and 0.23, respectively.

### Predictive performance of models

The residuals showed no strong evidence of a violation of the assumptions for linear regression for any of the models.

In general, all single blocks contributed to explained variance of all outcomes, and the relative contribution of the entered blocks of predictors were quite similar across outcomes (Additional file 2). The pain patterns (Block 1) and the baseline variable of the corresponding outcome measure (Block 5) explained most of the variance. Regarding the predictive ability of baseline values of the respective outcome measures (Block 5), baseline pain intensity contributed less to the explained variance compared to baseline NDI and EQ-5D. The single Block 2 (radiating pain and number of MSK pain-sites) and 3, (education level and physical leisure activity) had similar contribution to the explained variance across outcomes. The single Block 4 (consultation-type) did not contribute significantly to explained variance.

**Fig. 1:**
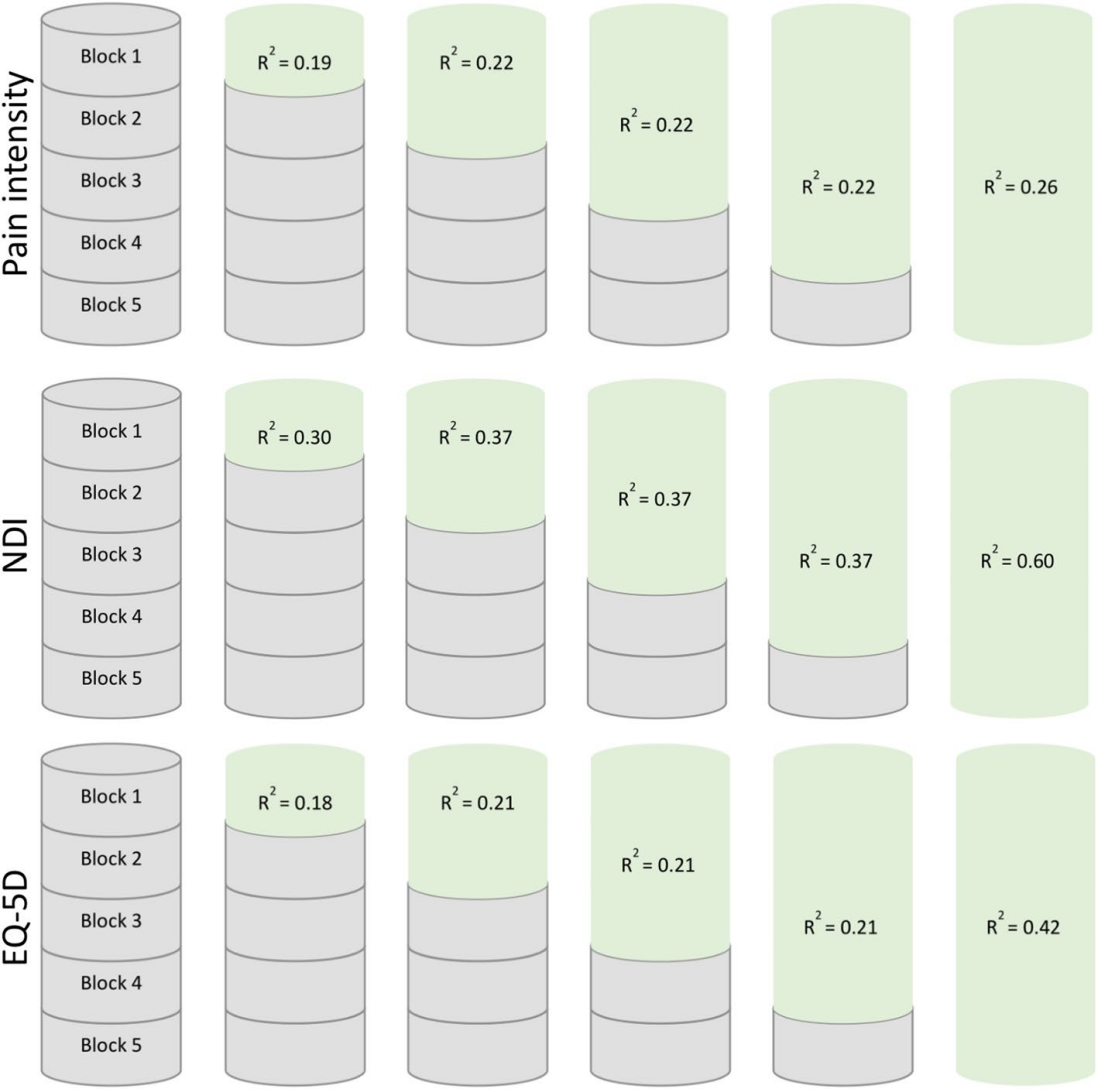
The predictive performance (Adjusted R^2^) when sequentially combining the five blocks for each outcome

Figure 1 and Tables 2, 3 and 4. presents the block-wise regression models with pain intensity, NDI and EQ-5D as outcomes, respectively. During the block-wise entry, the adjusted R^2^ values were largely unaltered from Block 1 until Block 4 for all outcomes. The final model having the highest explained variance, regardless of which baseline outcome variable. When we included the baseline variable of the corresponding outcome measure to the final model (Block 1 to 5), the adjusted R^2^ values ranged from 0.26 to 0.60 across outcomes. NDI was persistently the outcome with the highest explained variance compared to pain intensity and EQ-5D, with larger adjusted R^2^ values for single blocks as well as for the final model.

**Table 2:** The explained variance (Adjusted R^2^) and associations between pain intensity as outcome and predictors (entered in 5 blocks) explored by linear regression analysis, (*n* = 941)

**Blocks of predictors**		**Block 1**	**Block 1–2**	**Block 1–3**	**Block 1–4 (Final model)**	**Block 1-5a**
		**Adjusted R**^**2**^	**Adjusted R**^**2**^	**Adjusted R**^**2**^	**Adjusted R**^**2**^	**Adjusted R**^**2**^
		0.19	0.22	0.22	0.22	0.26
		**Coefficients (95% CI)**	**Coefficients (95% CI)**	**Coefficients (95% CI)**	**Coefficients (95% CI)**	**Coefficients (95% CI)**
**Patient previous course of pain** (n, %)						
Single episode	**Block 1**	Ref	Ref	Ref	Ref	Ref
Episodic pain		**6.66 (1.76 to 11.55)**	**5.16 (0.32 to 9.99)**	**5.10 (0.26 to 9.95)**	**5.14 (0.28 to 10.00)**	3.27 (-1.49 to 8.03)
Mild pain/recovering		**8.96 (2.57 to 14.82)**	**7.44 (1.40 to 13.49)**	**7.30 (1.24 to 13.37)**	**7.27 (1.20 to 13.35)**	5.71 (-0.21 to 11.63)
Fluctuating pain		**17.10 (12.14 to 22.06)**	**13.96 (8.98 to 18.94)**	**13.78 (8.79 to 18.78)**	**13.71 (8.71 to 18.71)**	**9.85 (4.86 to 14.83)**
Moderate/severe pain		**25.43 (16.12 to 34.74)**	**21.43 (12.21 to 30.66)**	**21.09 (11.83 to 30.35)**	**21.01 (11.74 to 30.29)**	**12.45 (3.09 to 21.81)**
Neither/ Unsure		**10.17 (1.16 to 19.17)**	**10.77 (1.84 to 19.70)**	**10.57 (1.63 to 19.52)**	**10.55 (1.60 to 19.51)**	8.37 (-0.21 to 16.95)
**Patient expected course of pain** (n, %)						
Single episode	**Block 2**	Ref	Ref	Ref	Ref	Ref
Episodic pain		**9.40 (5.71 to 13.10)**	**8.63 (5.00 to 12.56)**	**8.62 (4.98 to 12.25)**	**8.57 (4.91 to 12.23)**	**7.10 (3.50 to 10.69)**
Mild pain/recovering		**8.06 (3.11 to 13.01)**	**7.04 (2.17 to 11.91)**	**7.15 (2.26 to 12.04)**	**7.13 (2.21 to 12.04)**	**6.15 (1.32 to 10.99)**
Fluctuating pain		**12.57 (8.18 to 16.98)**	**11.07 (6.73 to 15.41)**	**10.91 (6.54 to 15.27)**	**10.94 (6.48 to 15.40)**	**9.52 (5.14 to 13.91)**
Moderate/severe pain		**28.84 (10.96 to 46.74)**	**27.22 (9.72 to 44.71)**	**27.80 (10.25 to 45.35)**	**27.71 (10.09 to 45.33)**	**24.07 (6.86 to 41.29)**
Neither/ Unsure		**7.52 (2.86 to 12.19)**	**6.22 (1.62 to 10.82)**	**6.26 (1.65 to 10.86)**	**6.26 (1.64 to 10.88)**	4.49 (-0.03 to 9.01)
**Radiating pain to shoulder and/or elbow** (Ref.: yes)			-0.50 (-3.60 to 2.60)	-0.35 (-3.47 to 2.77)	-0.31 (-3.43 to 2.81)	-1.54 (-4.59 to 1.51)
**Number of MSK pain sites**			**1.91 (1.28 to 2.55)**	**1.69 (0.66 to 2.73)**	**1.69 (0.66 to 2.72)**	**1.30 (0.29 to 2.31)**
**Education level**						
Low	**Block 3**			Ref	Ref	Ref
Medium				-3.27 (-8.82 to 2.27)	-3.24 (-8.79 to 2.32)	-3.28 (-8.68 to 2.13)
High				-3.22 (-8.70 to 2.27)	-3.20 (-8.69 to 2.29)	-3.52 (-8.86 to 1.82)
**Physical leisure activity** (Ref.: yes)				-1.61 (-7.67 to 4.44)	-1.57 (-7.64 to 4.49)	-2.74 (-8.87 to 3.19)
**Physical leisure activity#Number of MSK pain-sites**				0.29 (-0.88 to 1.45)	0.28 (-0.89 to 1.45)	0.46 (-0.68 to 1.61)
**Consultation-type**						
First-time consultation	**Block 4**				Ref	Ref
Follow-up consultation					1.60 (-2.09 to 5.29)	2.68 (-0.95 to 6.30)
Maintenance consultation					1.03 (-2.38 to 4.45)	3.13 (-0.27 to 6.53)
**Baseline pain intensity**	**Block 5a**					**2.23 (1.64 to 2.82)**

*Note*: The 95% C.I not including 0 are marked in bold, The NRS pain intensity is transformed to a 0–100 scale for both baseline and outcome

**Table 3:** The explained variance (Adjusted R^2^) and associations between disability as outcome and predictors (entered in 5 blocks) explored by linear regression analysis, (*n* = 939)

Blocks of predictors		Block 1	Block 1–2	Block 1–3	Block 1–4 (Final model)	Block 1-5b
		Adjusted R^2^	Adjusted R^2^	Adjusted R^2^	Adjusted R^2^	Adjusted R^2^
		0.30	0.37	0.37	0.37	0.60
		Coefficients (95% CI)	Coefficients (95% CI)	Coefficients (95% CI)	Coefficients (95% CI)	Coefficients (95% CI)
**Patient previous course of pain** (n, %)						
Single episode	**Block 1**	Ref	Ref	Ref	Ref	Ref
Episodic pain		**3.54 (0.71 to 6.37)**	2.25 (-0.48 to 4.97)	2.22 (-0.51 to 4.95)	2.21 (-0.52 to 4.94)	0.24 (-1.97 to 2.44)
Mild pain/recovering		**4.66 (1.06 to 8.26)**	**3.65 (0.19 to 7.12)**	3.46 (-0.02 to 6.94)	3.40 (-0.07 to 6.87)	1.97 (-0.83 to 4.78)
Fluctuating pain		**12.52 (9.64 to 15.40)**	**9.86 (7.04 to 12.67)**	**9.68 (6.85 to 12.50)**	**9.60 (6.78 to 12.43)**	**3.08 (0.71 to 5.44)**
Moderate/severe pain		**19.54 (14.17 to 24.92)**	**16.10 (10.94 to 21.26)**	**15.82 (10.64 to 21.00)**	**15.77 (10.59 to 20.96)**	**5.28 (1.01 to 9.55)**
Neither/ Unsure		**5.83 (0.74 to 10.92)**	**6.42 (1.54 to 11.30)**	**6.25 (1.36 to 11.14)**	**6.20 (1.31 to 11.09)**	2.66 (-1.27 to 6.60)
**Patient expected course of pain** (n, %)						
Single episode	**Block 2**	Ref	Ref	Ref	Ref	Ref
Episodic pain		**5.41 (3.29 to 7.54)**	**4.74 (2.72 to 6.77)**	**4.75 (2.72 to 6.77)**	**4.67 (2.63 to 6.70)**	**4.33 (2.69 to 5.96)**
Mild pain/recovering		**5.12 (2.26 to 7.98)**	**4.11 (1.38 to 6.84)**	**4.27 (1.53 to 7.00)**	**4.20 (1.45 to 6.94)**	**3.83 (1.63 to 6.02)**
Fluctuating pain		**8.35 (5.81 to 10.90)**	**7.00 (4.58 to 9.43)**	**6.93 (4.49 to 9.37)**	**6.86 (4.37 to 9.35)**	**5.60 (3.58 to 7.62)**
Moderate/severe pain		**27.06 (16.64 to 37.48)**	**25.62 (15.79 to 35.46)**	**25.97 (16.12 to 35.82)**	**25.75 (15.86 to 35.65)**	**13.73 (5.06 to 22.39)**
Neither/ Unsure		**4.88 (2.20 to 7.56)**	**3.64 (1.08 to 6.20)**	**3.64 (1.08 to 6.20)**	**3.61 (1.04 to 6.18)**	**3.34 (1.29 to 5.40)**
**Radiating pain to shoulder and/or elbow** (Ref.: yes)			-1.45 (-3.18 to 0.29)	-1.31 (-3.06 to 0.43)	-1.27 (-3.02 to 0.48)	-1.30 (-2.69 to 0.09)
**Number of MSK pain sites**			**1.79 (1.43 to 2.14)**	**1.44 (0.87 to 2.02)**	**1.44 (0.87 to 2.02)**	**0.56 (0.09 to 1.03)**
**Education level**						
Low	**Block 3**			Ref	Ref	Ref
Medium				-1.65 (-4.76 to 1.47)	-1.64 (-4.76 to 1.47)	-0.04 (-2.54 to 2.45)
High				-2.19 (-5.27 to 0.89)	-2.19 (-5.27 to 0.89)	-0.67 (-3.13 to 1.80)
**Physical leisure activity** (Ref.: yes)				-2.25 (-5.63 to 1.12)	-2.19 (-5.57 to 1.18)	-1.23 (-3.94 to 1.48)
** Physical leisure activity#Number of MSK pain-sites**				0.48 (-0.17 to 1.13)	0.47 (-0.19 to 1.12)	0.22 (-0.31 to 0.74)
**Consultation-type**						
First-time consultation	**Block 4**				Ref	Ref
Follow-up consultation					1.31 (-0.73 to 3.35)	**2.04 (0.39 to 3.68)**
Maintenance consultation					1.12 (-0.78 to 3.03)	**3.16 (1.62 to 4.70)**
**Baseline NDI**	**Block 5b**					**0.56 (0.51 to 0.61)**

*Note*: The 95% C.I not including 0 are marked in bold, The NDI is transformed to a 0–100 scale for both baseline and outcome

**Table 4:** The explained variance (Adjusted R^2^) and associations between EQ-5D as outcome and predictors (entered in 5 blocks) explored by linear regression analysis (n = 941)

**Blocks of predictors**		**Block 1**	**Block 1–2**	**Block 1–3**	**Block 1–4 (Final model)**	**Block 1-5c**
		**Adjusted R**^**2**^	**Adjusted R**^**2**^	**Adjusted R**^**2**^	**Adjusted R**^**2**^	**Adjusted R**^**2**^
		0.18	0.21	0.21	0.21	0.42
		**Coefficients (95% CI)**	**Coefficients (95% CI)**	**Coefficients (95% CI)**	**Coefficients (95% CI)**	**Coefficients (95% CI)**
**Patient previous course of pain** (n, %)						
Single episode	**Block 1**	Ref	Ref	Ref	Ref	Ref
Episodic pain		-1.69 (-4.24 to 0.86)	-0.96 (-3.47 to 1.55)	-0.86 (-3.36 to 1.64)	-0.85 (-3.36 to 1.67)	1.05 (-3.23 to 1.12)
Mild pain/recovering		-1.99 (-5.18 to 1.20)	-1.44 (-4.58 to 1.71)	-1.37 (-4.51 to 1.77)	-1.38 (-4.53 to 1.76)	-1.92 (-4.64 to 0.81)
Fluctuating pain		**-6.39 (-8.97 to -3.80)**	**-4.83 (-7.42 to -2.25)**	**-4.69 (-7.28 to -2.11)**	**-4.72 (-7.31 to -2.13)**	-3.06 (-5.30 to -0.81)
Moderate/severe pain		**-11.66 (-16.52 to -6.80)**	**-9.67 (-14.48 to -4.87)**	**-9.54 (-14.35 to -4.74)**	**-9.57 (-14.38 to -4.76)**	-2.42 (-6.65 to 1.81)
Neither/ Unsure		**-4.90 (-9.61 to -0.18)**	**-5.27 (-9.91 to -0.62)**	**-5.04 (-9.67 to -0.40)**	**-5.05 (-9.68 to -0.41)**	**-4.64 (-8.77 to -0.51)**
**Patient expected course of pain** (n, %)						
Single episode	**Block 2**	Ref	Ref	Ref	Ref	Ref
Episodic pain		**-3.09 (-5.02 to -1.17)**	**-2.68 (-4.57 to -0.79)**	**-2.69 (-4.57 to -0.80)**	**-2.70 (-4.60 to -0.80)**	**-2.07 (-3.70 to -0.43)**
Mild pain/recovering		**-3.77 (-6.35 to -1.18)**	**-3.14 (-5.68, -0.61)**	**-3.15 (-5.68 to -0.61)**	**-3.15 (-5.70 to -0.60)**	**-2.40 (-4.60 to -0.19)**
Fluctuating pain		**-6.25 (-8.55 to -3.96)**	**-5.45 (-7.71 to -3.19)**	**-5.39 (-7.66 to -3.13)**	**-5.38 (-7.70 to -3.06)**	**-4.12 (-6.12 to -2.11)**
Moderate/severe pain		**-25.32 (-34.65 to -16.00)**	**-24.47 (-33.62 to -15.32)**	**-25.36 (-34.50 to -16.22)**	**-25.39 (-34.58 to -16.21)**	**-21.59 (-29.51 to -13.66)**
Neither/ Unsure		**-2.67 (-5.10 to -0.23)**	-1.91 (-4.30 to 0.48)	-1.98 (-4.37 to 0.40)	-1.98 (-4.38 to 0.42)	-1.50 (-3.57 to 0.58)
**Radiating pain to shoulder and/or elbow** (Ref.: yes)			1.23 (-0.37 to 2.84)	1.15 (-0.46 to 2.77)	1.16 (-0.45 to 2.78)	1.14 (-0.27 to 2.55)
**Number of MSK pain sites**			**-1.09 (-1.42 to -0.76)**	**-0.91 (-1.44 to -0.37)**	**-0.91 (-1.44 to -0.37)**	-0.23 (-0.70 to 0.24)
**Education level**						
Low	**Block 3**			Ref	Ref	Ref
Medium				**3.56 (0.47 to 6.25)**	**3.37 (0.48 to 6.27)**	1.50 (-1.01 to 4.00)
High				2.21 (-0.66 to 5.07)	2.21 (-0.65 to 5.08)	1.04 (-1.43 to 3.51)
**Physical leisure activity** (Ref.: yes)				2.06 (-1.08 to 5.20)	2.07 (-1.07 to 5.22)	2.02 (-0.69 to 4.73)
**Physical leisure activity#Number of MSK pain-sites**				-0.25 (-0.86 to 0.35)	-0.26 (-0.86 to 0.35)	-0.35 (-0.88 to 0.17)
**Consultation-type**						
First-time consultation	**Block 4**				Ref	Ref
Follow-up consultation					0.56 (-1.34 to 2.46)	-0.85 (-2.50 to 0.80)
Maintenance consultation					0.36 (-1.40 to 2.13)	**-1.88 (-3.43 to -0.34)**
**Baseline EQ-5D**	**Block 5c**					**0.41 (0.37 to 0.46)**

*Note*: The 95% C.I not including 0 are marked in bold, The EQ5D index-scale is transformed to a 0–100 scale for both baseline and outcome

The impact of the baseline values for different outcomes were further explored in Table 5. Adding baseline values for NDI to the final model (Block 1–4) resulted in the largest increase in adjusted R^2^ across all outcomes. Baseline pain intensity had little impact even when pain intensity was the outcome.

**Table 5:** The explained variance (Adjusted R^2^) between baseline outcome variables, predictors and NDI, EQ-5D or pain intensity as outcome after 12 weeks explored by linear regression analysis

	**Outcomes**		
	**Pain intensity**	**NDI**	**EQ-5D**
	*n* = 941	*n* = 939	*n* = 941
**Baseline outcome variables/Model**	**Adjusted R**^**2**^	**Adjusted R**^**2**^	**Adjusted R**^**2**^
**Baseline pain intensity** (only Block 5a)	0.11	0.11	0.05
**Baseline NDI** (only Block 5b)	0.19	0.53	0.26
**Baseline EQ-5D** (only Block 5c)	0.11	0.26	0.34
**Final model** (Block 1 to 4), **no baseline variable**	0.22	0.37	0.21
**Final model and baseline pain intensity** (Block 1 to 5a)	0.25	0.39	0.22
**Final model and baseline NDI** (Block 1 to 5b)	0.27	0.60	0.31
**Final model and baseline EQ-5D** (Block 1 to 5c)	0.25	0.47	0.42

*Note*: The scales are transformed to a 0–100 scale for both baseline and outcome

### Predictors in the models

In the final models (including Block 5), the pain patterns (Block 1) and the corresponding outcome variable (Block 5) were significantly associated with all outcomes (Tables 2, 3 and 4). Of the other predictors, consultation-type (Block 4) significantly associated with NDI and EQ-5D as outcomes. Number of pain sites were significantly associated with pain intensity and NDI as outcome. For all outcomes, the association with of education level, radiating pain to the shoulder and/or elbow and physical leisure activity was weak. The 95% CI were large for all predictors across outcomes; thus, comparisons should be interpreted with caution.

## Discussion

This study found weak to moderate associations between improvements on the scales of pain intensity, NDI and EQ-5D outcome instruments. We also found that the prognostic model developed for the prediction of GPE showed large differences in total explained variance across three outcomes (pain intensity, NDI and EQ-5D). The prognostic model showed poorer predictive ability for pain intensity compared to both NDI and EQ-5D. For all outcomes, the impact of the individual entered blocks of predictors were quite similar. Among the investigated predictors, pain patterns accounted for the largest explained variance in all outcomes.

Our results show that with the chosen set of predictors, disability is more accurately predicted than pain intensity and EQ-5D. The difference in explained variance between pain intensity and NDI is in keeping with previous studies using pain intensity and disability as outcomes [19, 21, 22, 45]. One study included a large number of psychological candidate prognostic factors [21], while another more recent study included a wide selection of biopsychosocial candidate predictors [22]. Hence, it is reasonable to suggest that the consistent differences in performance with different outcomes, is due to measurement properties of the various outcomes.

One possible explanation for the lower performance of the prognostic model using pain intensity as outcome may be due to the poor reliability of a single pain measure [46, 47]. As neck pain is reported to be episodic or fluctuating [48–50], it is less likely that an improvement of symptoms is captured by one single time point measurement on a group level. The phenomena captured by the composite measures (e.g., NDI and EQ-5D) might be less subject to such variation over time. Another explanation may be construct differences of outcome measures [47]. Multidimensional constructed outcomes may better capture the complexity of the individual’s experience of neck pain compared to single dimension outcomes such as pain intensity [47, 51].

MSK patients emphasize pain as an important goal and pain is commonly evaluated in clinical practice [2]. Accordingly, randomized clinical trials use pain intensity as an outcome. Although it seems relevant to assess pain, the poorer ability to capture improvement may lead to weaknesses in the trials. This is a challenge that needs attention and further studies for instance through exploring why changes in pain is difficult to predict and how pain intensity can be used best as an outcome measure.

The lower correlation between pain intensity and either of the two other outcomes, than between NDI and EQ-5D further supports that pain intensity is either less reliable or have a different construct. Although pain intensity is a simple one item measure, pain affliction may be modified by numerous other factors like expectations, pain beliefs and behaviors [52, 53]. The more concrete items included in NDI and EQ-5D may be less vulnerable to such modulation, and some of the differences may possibly be related to this effect.

Independent of outcome measure, the pain patterns and baseline values of outcomes were the predictors that contributed the most to prediction. Previously, neck pain history (including previous episodes and duration) and future expectations assessed by traditional measures (i.e., numeric scales) have been found to be predictors of GPE, pain and disability [17, 19, 22, 30, 45]. These findings support our results on pain history and expectations as consistent prognostic factors. Similar to systematic reviews on prognostic factors, we also found baseline pain intensity and disability as robust predictors of outcomes [14, 16].

Although it can be argued that there is an implicit perception that pain intensity is included in the pain patterns (Block 1), we found that pain intensity as a single predictor or when added to the final model only contributes little to prediction, regardless of outcome measure. The association with outcome for the remaining predictors (radiating pain to shoulder and/or elbow, physical leisure activity, MSK pain-sites and education level) were less consistent, in line with previous research on prognostic factors and models for neck pain [18, 20, 22]. Consultation-type has previous proven to be associated with outcome [20], but not to interact with other predictors [20, 30]. A recent study used a set of predefined prognostic factors to develop prognostic models for recovery of patients with neck pain [22]. This study used pain, disability, and perceived effect as outcomes. Like us, they found the prognostic model with disability as outcome to have the best predictive performance but found only neck pain duration as a consistent predictor across all outcomes. This emphasises that a prognostic factor or model derived for a specific outcome does not fully represent all health domains, and thus models need validation on the outcome they are intended for.

### Strengths and limitations

One limitation concerns the loss to follow-up. Since 28% of the included patients did not respond at 12 weeks follow-up a possibility of attrition bias exits. The main reason to not answer the questionnaire at 12 weeks follow-up was due to lack of time required to complete questionnaires. However, the non-responders did not differ significantly from the analyzed sample suggesting that a possible impact of attrition bias may not have substantial influenced the results. Another concern is that the inclusion criteria to own a mobile phone and to adequate understand the Norwegian language may introduce selection bias. The most common reasons were ‘patient did not wish to participate’ followed by ‘chiropractor forgot or did not have time to ask’, thus we believe the risk of selection bias due to these parameters is low. The included predictors were derived using the single item GPE as outcome in a previous study [20]. We were therefore unable to directly compare results to other studies because there is no obvious way of transforming GPE to a comparable scale. However, as GPE do not represent a specific health domain, GPE as outcome did not favor any of the investigated outcomes. A strength is that the inclusion criteria were broad rather than strict, as in RCT’s, and this can result in a more heterogeneous patient population. Consequently, this may support the generalization of the results to patients with neck pain seen in primary health care. We included consultation-type because the participating patients were not included at a uniform time (zero time). Patients that seek care across health care settings experience different phases of their neck pain course (i.e., acute, recurring, or persistent), which is a challenge for clinicians regarding prognosis. Therefore, in the regression analyses of this study, we considered for these differences at inception by adding the variable consultation-type. We found that consultation-type did not interact with the included prognostic factors, but it seems that the inception time is related to outcome. Although not useful on an individual level, consultation-type may be one way to achieve additional prognostic information for a setting where the patient population are heterogeneous. The Visual trajectory pattern questionnaire has not been validated. However, it is quite similar to a questionnaire used for low back pain that seem to capture peoples prospectively measured course [29]. Also, the relationship with patient reported outcomes in the expected direction provides support for the responses to the questionnaire to be meaningful.

## Conclusions

The highest correlation between outcome change scores was found between NDI and EQ-5D and lower association with pain. The prognostic model also showed best performance for NDI as outcome and the poorest for pain intensity. The predictive impact of the predictors was consistent across all outcomes. These results suggest that we need more knowledge on the reasons for the differences in predictive performance variation across outcomes.

## Supplementary Information

**Additional file 1: Figure. **Lowess plots for pain intensity, NDI and EQ-5D outcome change scores. (A) NDI and pain intensity. (B) Pain intensity and EQ-5D. (C) NDI and EQ-5D. 

**Additional file 2:** **Table.** The explained variance (Adjusted R^2^) and parametersbetween each single block and pain intensity, NDI and EQ-5D as outcome after 12weeks explored by linear regression analysis.

**Additional file 3: Figure. **Flowchart showing the participation in the study including thedata set used for analysis in the study. 

## Data Availability

The data that supports the findings of the current study are not publicly available due to data protection policies. Data are however available from the corresponding author on reasonable request.

## Abbreviations

HRQoL: Health-Related Quality of Life
GPE: Global Perceived Effect
MSK: Musculoskeletal
NDI: The Neck Disability Index questionnaire
SD: Standard deviation
IQR: Interquartile range
95% CI: 95% Confidence interval

## References

[1] Turk DC, Dworkin RH, Revicki D, Harding G, Burke LB, Cella D (2008). Identifying important outcome domains for chronic pain clinical trials: an IMMPACT survey of people with pain. Pain.

[2] Henry SG, Bell RA, Fenton JJ, Kravitz RL (2017). Goals of chronic pain management: Do patients and primary care physicians agree and does it matter?. Clin J Pain.

[3] Gardner T, Refshauge K, McAuley J, Goodall S, Hubscher M, Smith L (2016). Patient-led Goal Setting: A pilot study investigating a promising approach for the management of chroniclLow back pain. Spine.

[4] Picavet HS, Schouten JS (2003). Musculoskeletal pain in the Netherlands: prevalences, consequences and risk groups, the DMC(3)-study. Pain.

[5] Amundsen O, Vollestad NK, Meisingset I, Robinson HS (2021). Associations between treatment goals, patient characteristics, and outcome measures for patients with musculoskeletal disorders in physiotherapy practice. BMC Musculoskelet Disord.

[6] Zeppieri G, Bialosky J, George SZ (2020). Importance of outcome domain for patients with musculoskeletal pain: Characterizing subgroups and their response to treatment. Phys Ther.

[7] Bobos P, MacDermid JC, Walton DM, Gross A, Santaguida PL (2018). Patient-Reported Outcome Measures used for neck disorders: An overview of systematic reviews. J Orthop Sports Phys Ther.

[8] Macdermid JC, Walton DM, Cote P, Santaguida PL, Gross A, Carlesso L (2013). Use of outcome measures in managing neck pain: an international multidisciplinary survey. Open Orthop J.

[9] Turk DC, Dworkin RH, Allen RR, Bellamy N, Brandenburg N, Carr DB (2003). Core outcome domains for chronic pain clinical trials: IMMPACT recommendations. Pain.

[10] Carreon LY, Bratcher KR, Das N, Nienhuis JB, Glassman SD (2014). Estimating EQ-5D values from the Neck Disability Index and numeric rating scales for neck and arm pain. J Neurosurg Spine.

[11] Chiu TT, Lam TH, Hedley AJ (2005). Correlation among physical impairments, pain, disability, and patient satisfaction in patients with chronic neck pain. Arch Phys Med Rehabil.

[12] DeVine J, Norvell DC, Ecker E, Fourney DR, Vaccaro A, Wang J (2011). Evaluating the correlation and responsiveness of patient-reported pain with function and quality-of-life outcomes after spine surgery. Spine.

[13] Hemingway H, Croft P, Perel P, Hayden JA, Abrams K, Timmis A (2013). Prognosis research strategy (PROGRESS) 1: a framework for researching clinical outcomes. BMJ (Clinical research ed).

[14] Mansell G, Corp N, Wynne-Jones G, Hill J, Stynes S, van der Windt D (2021). Self-reported prognostic factors in adults reporting neck or low back pain: An umbrella review. Eur J Pain.

[15] Green DJ, Lewis M, Mansell G, Artus M, Dziedzic KS, Hay EM (2018). Clinical course and prognostic factors across different musculoskeletal pain sites: A secondary analysis of individual patient data from randomised clinical trials. Eur J Pain.

[16] Walton DM, Carroll LJ, Kasch H, Sterling M, Verhagen AP, Macdermid JC (2013). An Overview of systematic reviews on prognostic factors in neck pain: Results from the International Collaboration on Neck Pain (ICON) Project. Open Orthop J.

[17] Bishop MD, Mintken PE, Bialosky JE, Cleland JA (2013). Patient expectations of benefit from interventions for neck pain and resulting influence on outcomes. J Orthop Sports Phys Ther.

[18] Verwoerd M, Wittink H, Maissan F, de Raaij E, Smeets R (2019). Prognostic factors for persistent pain after a first episode of nonspecific idiopathic, non-traumatic neck pain: A systematic review. Musculoskelet Sci Pract.

[19] Wingbermuhle RW, van Trijffel E, Nelissen PM, Koes B, Verhagen AP (2018). Few promising multivariable prognostic models exist for recovery of people with non-specific neck pain in musculoskeletal primary care: a systematic review. J Physiother.

[20] Myhrvold BL, Kongsted A, Irgens P, Robinson HS, Thoresen M, Vollestad NK. Broad external validation and update of a prediction model for persistent neck pain after 12 weeks. Spine. 2019;44(22):E1298–310. 10.1097/BRS.0000000000003144 31689251

[21] Pool JJ, Ostelo RW, Knol D, Bouter LM, de Vet HC (2010). Are psychological factors prognostic indicators of outcome in patients with sub-acute neck pain?. Man Ther.

[22] Wingbermuhle RW, Chiarotto A, van Trijffel E, Koes B, Verhagen AP, Heymans MW (2021). Development and internal validation of prognostic models for recovery in patients with non-specific neck pain presenting in primary care. Physiotherapy.

[23] von Elm E, Altman DG, Egger M, Pocock SJ, Gotzsche PC, Vandenbroucke JP (2007). The Strengthening the Reporting of Observational Studies in Epidemiology (STROBE) statement: guidelines for reporting observational studies. Epidemiology.

[24] Vasseljen O, Woodhouse A, Bjorngaard JH, Leivseth L (2013). Natural course of acute neck and low back pain in the general population: the HUNT study. Pain.

[25] Nyiro L, Peterson CK, Humphreys BK (2017). Exploring the definition of neck pain: a prospective cohort observational study comparing the outcomes of chiropractic patients with 0–2 weeks, 2–4 weeks and 4–12 weeks of symptoms. Chiropr Man Therap.

[26] Kongsted A, Kent P, Hestbaek L, Vach W. Patients with low back pain had distinct clinical course patterns that were typically neither complete recovery nor constant pain. A latent class analysis of longitudinal data. Spine J. 2015;15(5):885–94.http://dx.doi.org/10.1016/j.spinee.2015.02.012 10.1016/j.spinee.2015.02.012 25681230

[27] Schellingerhout JM, Heymans MW, Verhagen AP, Lewis M, de Vet HC, Koes BW (2010). Prognosis of patients with nonspecific neck pain: development and external validation of a prediction rule for persistence of complaints. Spine.

[28] Kongsted A, Kent P, Axen I, Downie AS, Dunn KM (2016). What have we learned from ten years of trajectory research in low back pain?. BMC Musculoskelet Disord.

[29] Dunn KM, Campbell P, Jordan KP (2017). Validity of the Visual Trajectories Questionnaire for Pain. J Pain.

[30] Myhrvold BL, Irgens P, Robinson HS, Engebretsen K, Natvig B, Kongsted A (2020). Visual trajectory pattern as prognostic factors for neck pain. Eur J Pain.

[31] Myburgh C, Brandborg-Olsen D, Albert H, Hestbaek L (2013). The Nordic maintenance care program: what is maintenance care? Interview based survey of Danish chiropractors. Chiropr Man Therap.

[32] Von Korff M, Jensen MP, Karoly P (2000). Assessing global pain severity by self-report in clinical and health services research. Spine.

[33] Vernon H, Mior S (1991). The Neck Disability Index: a study of reliability and validity. J Manipulative Physiol Ther.

[34] Johansen JB, Andelic N, Bakke E, Holter EB, Mengshoel AM, Roe C (2013). Measurement properties of the Norwegian version of the Neck Disability Index in chronic neck pain. Spine.

[35] Vernon H (2008). The Neck Disability Index: state-of-the-art, 1991–2008. J Manipulative Physiol Ther.

[36] Schellingerhout JM, Verhagen AP, Heymans MW, Koes BW, de Vet HC, Terwee CB (2012). Measurement properties of disease-specific questionnaires in patients with neck pain: a systematic review. Qual Life Res.

[37] Brooks R (1996). EuroQol: the current state of play. Health Policy.

[38] Solberg TK, Olsen JA, Ingebrigtsen T, Hofoss D, Nygaard OP. Health-related quality of life assessment by the EuroQol-5D can provide cost-utility data in the field of low-back surgery. European spine journal : official publication of the European Spine Society, the European Spinal Deformity Society, and the European Section of the Cervical Spine Research Society. 2005;14(10):1000–7.http://dx.doi.org/10.1007/s00586-005-0898-2 10.1007/s00586-005-0898-2 15843969

[39] Wahlberg M, Zingmark M, Stenberg G, Munkholm M (2021). Rasch analysis of the EQ-5D-3L and the EQ-5D-5L in persons with back and neck pain receiving physiotherapy in a primary care context. European Journal of Physiotherapy.

[40] Mukaka MM (2012). Statistics corner: A guide to appropriate use of correlation coefficient in medical research. Malawi Med J.

[41] Schober P, Boer C, Schwarte LA (2018). Correlation coefficients: Appropriate use and interpretation. Anesth Analg.

[42] Cohen J. Statistical power analysis for the behavioral sciences: Routledge; 2013.

[43] Collins GS, Reitsma JB, Altman DG, Moons KGM. Transparent Reporting of a multivariable prediction model for Individual Prognosis Or Diagnosis (TRIPOD): the TRIPOD Statement. BMC medicine. 2015;13:1-.http://dx.doi.org/10.1186/s12916-014-0241-z 10.1186/s12916-014-0241-z PMC4284921 25563062

[44] Riley RD, Ensor J, Snell KIE, Harrell FE, Martin GP, Reitsma JB (2020). Calculating the sample size required for developing a clinical prediction model. BMJ (Clinical research ed).

[45] Hoving JL, de Vet HCW, Twisk JWR, Deville W, van der Windt D, Koes BW (2004). Prognostic factors for neck pain in general practice. Pain.

[46] Jensen MP, Tome-Pires C, Sole E, Racine M, Castarlenas E, de la Vega R (2015). Assessment of pain intensity in clinical trials: individual ratings vs composite scores. Pain Med.

[47] Chiarotto A, Maxwell LJ, Ostelo RW, Boers M, Tugwell P, Terwee CB (2019). Measurement properties of Visual Analogue Scale, Numeric Rating Scale, and Pain Severity Subscale of the Brief Pain Inventory in patients with low back pain: A systematic review. J Pain.

[48] Ailliet L, Rubinstein SM, Hoekstra T, van Tulder MW, de Vet HCW (2018). Long-term trajectories of patients with neck pain and low back pain presenting to chiropractic care: A latent class growth analysis. Eur J Pain.

[49] Pico-Espinosa OJ, Cote P, Hogg-Johnson S, Jensen I, Axen I, Holm LW (2019). Trajectories of pain intensity over 1 year in adults with disabling subacute or chronic neck pain. Clin J Pain.

[50] Irgens P, Kongsted A, Myhrvold BL, Waagan K, Engebretsen KB, Natvig B (2020). Neck pain patterns and subgrouping based on weekly SMS-derived trajectories. BMC Musculoskelet Disord.

[51] Hush JM, Refshauge K, Sullivan G, De Souza L, Maher CG, McAuley JH (2009). Recovery: what does this mean to patients with low back pain?. Arthritis Rheum.

[52] Hush JM, Refshauge KM, Sullivan G, De Souza L, McAuley JH (2010). Do Numerical Rating Scales and the Roland-Morris Disability Questionnaire capture changes that are meaningful to patients with persistent back pain?. Clin Rehabil.

[53] Robinson-Papp J, George MC, Dorfman D, Simpson DM (2015). Barriers to chronic pain measurement: A qualitative study of patient perspectives. Pain Med.

[54] Grotle M, Vollestad NK, Brox JI (2006). Screening for yellow flags in first-time acute low back pain: reliability and validity of a Norwegian version of the Acute Low Back Pain Screening Questionnaire. Clin J Pain.

[55] Linton SJ, Nicholas M, MacDonald S (2011). Development of a short form of the Orebro Musculoskeletal Pain Screening Questionnaire. Spine.

[56] Derogatis LR, Lipman RS, Rickels K, Uhlenhuth EH, Covi L (1974). The Hopkins Symptom Checklist (HSCL): a self-report symptom inventory. Behav Sci.

