# Supplementary material for: The association between different outcome measures and prognostic factors in patients with neck pain: a cohort study
Source: BMC Musculoskelet Disord. 2022 Jul 14;23:673. doi: 10.1186/s12891-022-05558-5 (PMC9281081; doi:10.1186/s12891-022-05558-5)
Supplement: Supplementary file 1 — Additional file 1: Figure. Lowess plots for pain intensity, NDI and EQ-5D outcome change scores. (A) NDI and pain intensity. (B) Pain intensity and EQ-5D. (C) NDI and EQ-5D. [file 12891_2022_5558_MOESM1_ESM.pdf]

Additional file 1 Figure. Lowess plots for pain intensity, NDI and EQ-5D outcome change scores. (A) NDI and pain intensity. (B) Pain intensity and EQ-5D. (C) NDI and EQ-5D

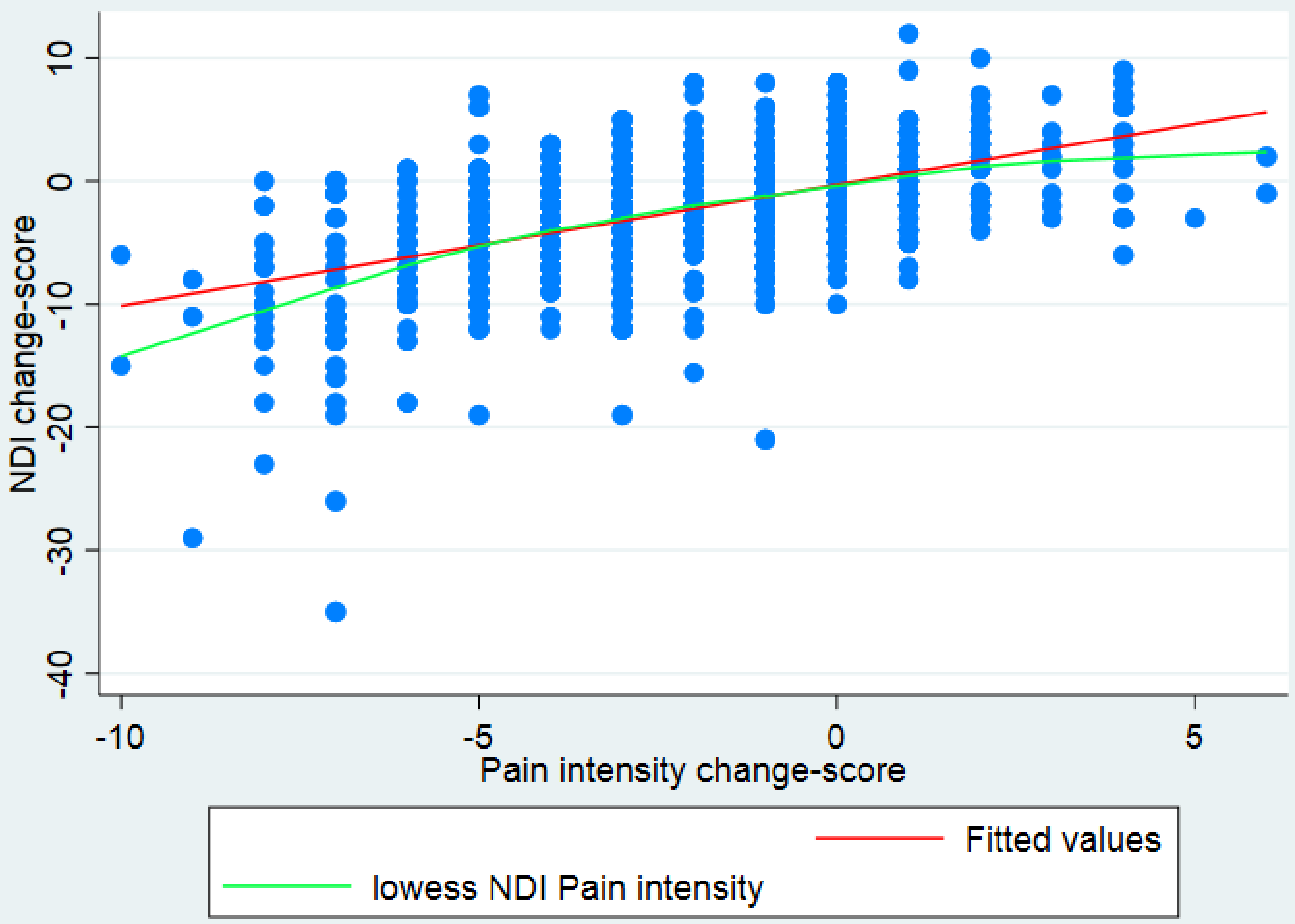

NDI change-score
10
0
-10
-20
-30
-40
-10
-5
0
5
Pain intensity change-score
Fitted values
lowess NDI Pain intensity

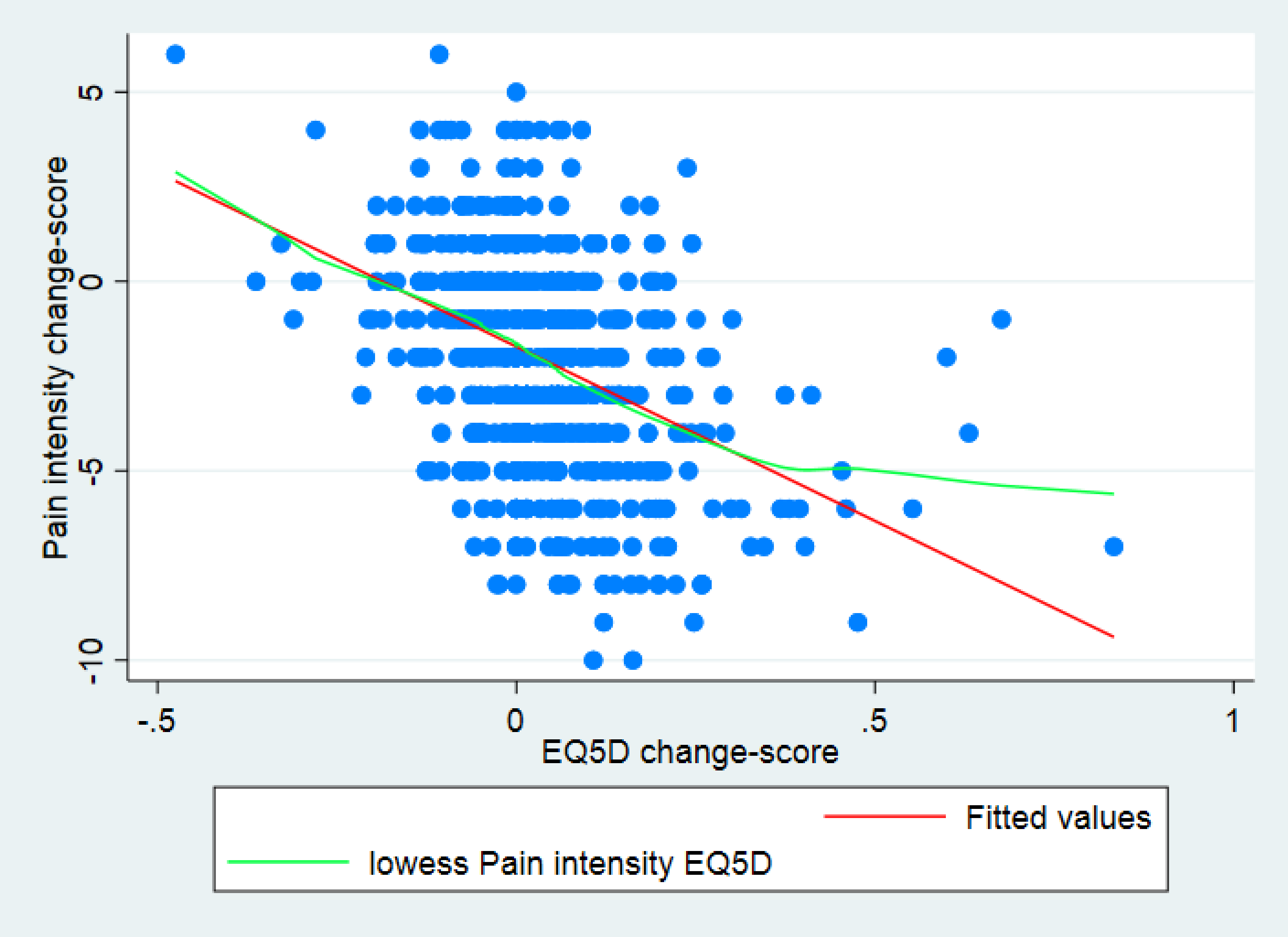

Pain intensity change-score
5
0
-5
-10
-.5
0
.5
1
EQ5D change-score
Fitted values
lowess Pain intensity EQ5D

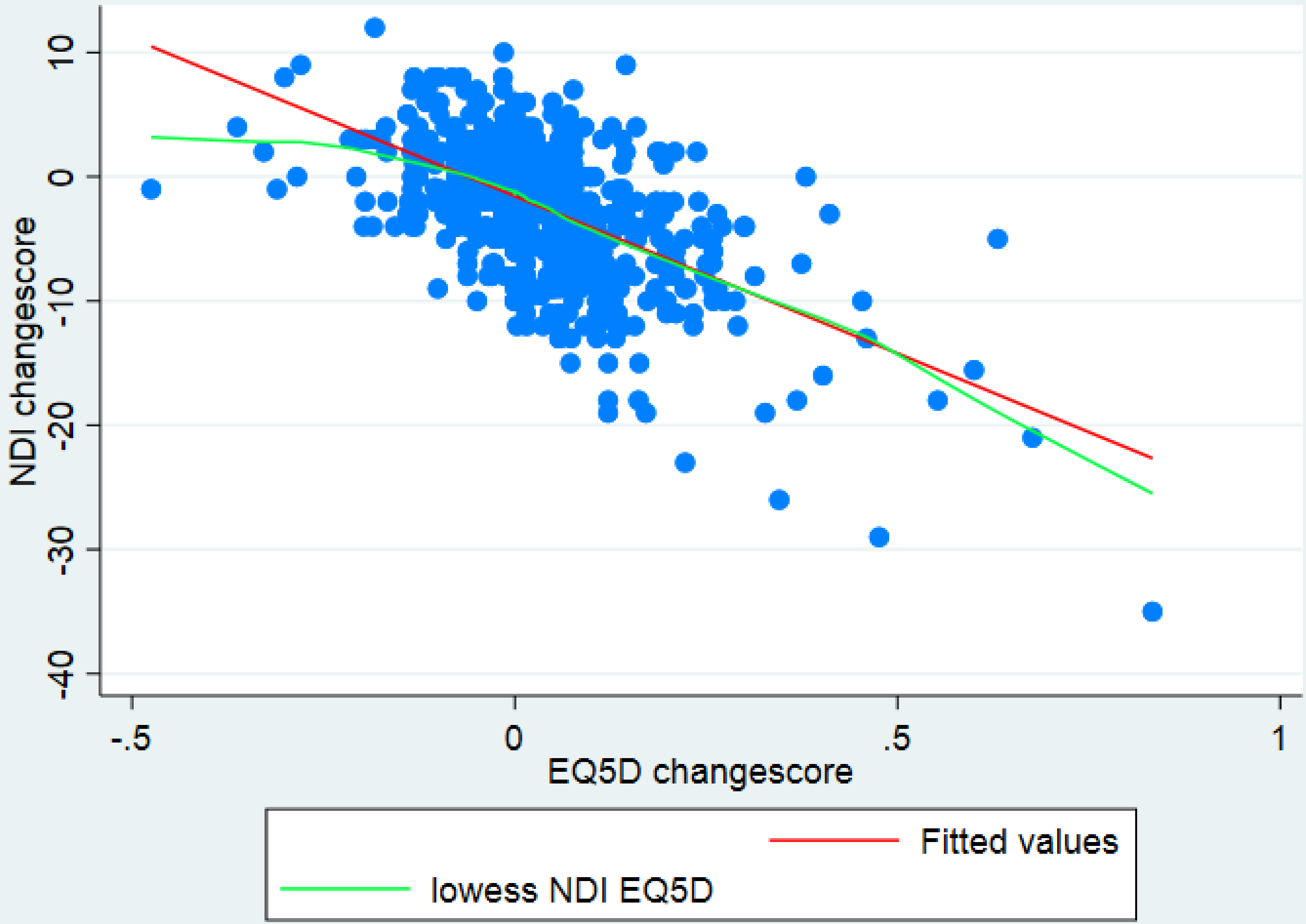
NDI changescore
10
0
-10
-20
-30
-40
-.5
0
.5
1
EQ5D changescore
Fitted values
lowess NDI EQ5D
