# Supplementary material for: The association between different outcome measures and prognostic factors in patients with neck pain: a cohort study
Source: BMC Musculoskelet Disord. 2022 Jul 14;23:673. doi: 10.1186/s12891-022-05558-5 (PMC9281081; doi:10.1186/s12891-022-05558-5)
Supplement: Supplementary file 2 — Additional file 2: Table. The explained variance (Adjusted R2) and parametersbetween each single block and pain intensity, NDI and EQ-5D as outcome after 12weeks explored by linear regression analysis. [file 12891_2022_5558_MOESM2_ESM.docx]

Additional file 2 Table. The explained variance (Adjusted R^2^) and parameters between each single block and pain intensity, NDI and EQ-5D as outcome after 12 weeks explored by linear regression analysis

|  |  | **Outcomes** | | |
| --- | --- | --- | --- | --- |
|  |  | **Pain intensity** | **NDI** | **EQ-5D** |
| Blocks of predictors |  | **Coefficients**  **(95% CI)** | **Coefficients**  **(95% CI)** | **Coefficients**  **(95% CI)** |
| **Patient previous course of pain** (n, %) | **Block 1** |  |  |  |
| Single episode |  | Ref. | Ref. | Ref. |
| Episodic pain |  | **6.66 (1.76 to 11.55)** | **3.54 (0.71 to 6.37)** | -1.69 (-4.24 to 0.86) |
| Mild pain/recovering |  | **8.96 (2.57 to 14.82)** | **4.66 (1.06 to 8.26)** | -1.99 (-5.18 to 1.20) |
| Fluctuating pain |  | **17.10 (12.14 to 22.06)** | **12.52 (9.64 to 15.40)** | **-6.39 (-8.97 to -3.80)** |
| Moderate/severe pain |  | **25.43 (16.12 to 34.74)** | **19.54 (14.17 to 24.92)** | **-11.66 (-16.52 to -6.80)** |
| Neither/ Unsure |  | **10.17 (1.16 to 19.17)** | **5.83 (0.74 to 10.92)** | **-4.90 (-9.61 to -0.18)** |
| **Patient expected course of pain** (n, %) |  |  |  |  |
| Single episode |  | Ref. | Ref. | Ref. |
| Episodic pain |  | **9.40 (5.71 to 13.10)** | **5.41 (3.29 to 7.54)** | **-3.09 (-5.02 to -1.17)** |
| Mild pain/recovering |  | **8.06 (3.11 to 13.01)** | **5.12 (2.26 to 7.98)** | **-3.77 (-6.35 to -1.18)** |
| Fluctuating pain |  | **12.57 (8.18 to 16.98)** | **8.35 (5.81 to 10.90)** | **-6.25 (-8.55 to -3.96)** |
| Moderate/severe pain |  | **28.84 (10.96 to 46.74)** | **27.06 (16.64 to 37.48)** | **-25.32 (-34.65 to -16.00)** |
| Neither/ Unsure |  | **7.52 (2.86 to 12.19)** | **4.88 (2.20 to 7.56)** | **-2.67 (-5.10 to -0.23)** |
| **Adjusted R^2^** |  | 0.19 | 0.30 | 0.18 |
| **Radiating pain** (Ref.: yes) | **Block 2** | 0.51 (-2.83 to 3.84) | -0.70 (-2.68 to 1.28) | 0.80 (-0.94 to 2.54) |
| **Number of MSK pain sites** |  | **3.03 (2.38 to 3.68)** | **2.62 (2.24 to 3.01)** | **-1.59 (-1.93 to -1.25)** |
| **Adjusted R^2^** |  | 0.10 | 0.19 | 0.09 |
| **Education level** | **Block 3** |  |  |  |
| Low |  | Ref. | Ref. | Ref. |
| Medium |  | **-6.47 (-12.35 to -0.58)** | **-4.10 (-7.58 to -0.62)** | **4.90 (1.86 to 7.94)** |
| High |  | **-7.65 (-13.43 to -1.87)** | **-5.73 (-9.15 to -2.32)** | **4.53 (1.54 to 7.51)** |
| **Physical leisure activity** (Ref.: yes) |  | -1.20 (-7.65 to 5.25) | -1.88 (-5.67 to 1.92) | 1.55 (-1.79 to 4.89) |
| **Physical leisure activity#Number of MSK pain-sites** |  |  |  |  |
| 0 |  | **2.87 (1.84 to 3.90)** | **2.23 (1.63 to 2.84)** | **-1.38 (-1.92 to -0.84)** |
| 1 |  | **3.08 (2.38 to 3.78)** | **2.64 (2.23 to3.06** | **-1.54 (-1.90 to -1.18)** |
| **Adjusted R^2^** |  | 0.10 | 0.19 | 0.10 |
| **Consultation-type** | **Block 4** |  |  |  |
| First-time consultation |  | Ref. | Ref. | Ref. |
| Follow-up consultation |  | 3.69 (-0.48 to 7.86) | **3.06 (0.52 to 5.61)** | -0.51 (-2.65 to 1.63) |
| Maintenance consultation |  | **5.20 (1.42 to 8.98)** | **4.24 (1.91 to 6.58)** | -1.59 (-3.54 to -0.36) |
| **Adjusted R^2^** |  | 0.005 | 0.01 | 0.002 |
| **Baseline variable** | **Block 5** | **0.29 (0.23 to 0.34)** | **0.70 (0.65 to 0.74)** | **0.48 (0.44 to 0.52)** |
| **Adjusted R^2^** |  | 0.11 | 0.53 | 0.34 |
