# Supplementary material for: The association between different outcome measures and prognostic factors in patients with neck pain: a cohort study
Source: BMC Musculoskelet Disord. 2022 Jul 14;23:673. doi: 10.1186/s12891-022-05558-5 (PMC9281081; doi:10.1186/s12891-022-05558-5)
Supplement: Supplementary file 3 — Additional file 3: Figure. Flowchart showing the participation in the study including thedata set used for analysis in the study. [file 12891_2022_5558_MOESM3_ESM.docx]

Additional file 3 Figure. Flowchart showing the participation in the study including the data set used for analysis in the study

Imputed data set for analysis

n=941

Data set containing missing baseline data

n=897

Lost to follow-up

Non-responders (n=372, 28%)

Missing baseline data for analysis

Previous pattern (n=8, 0.9%)

Expected pattern (n=1, 0.1%)

Radiating pain to shoulder and/or elbow (n=11, 1.2%)

Education level (n=1, 0.1%)

Physical leisure activity (n=1, 0.1%)

Consultation type (n=27, 2.9%)

Baseline pain intensity (n=9, 1.0%)

Baseline NDI (n=12, 1.3%)

Total study sample

n=941

Patients assessed for eligible

n=1313
